# The Influence of Personality, Resilience, and Alexithymia on Mental Health During COVID-19 Pandemic

**DOI:** 10.3389/fpsyg.2021.630751

**Published:** 2021-02-24

**Authors:** Sofia Adelaide Osimo, Marilena Aiello, Claudio Gentili, Silvio Ionta, Cinzia Cecchetto

**Affiliations:** ^1^Sensory-Motor Lab (SeMoLa), Department of Ophthalmology, Jules Gonin Eye Hospital-Fondation Asile des Aveugles, University of Lausanne, Lausanne, Switzerland; ^2^Scuola Internazionale Superiore di Studi Avanzati, Trieste, Italy; ^3^Department of General Psychology, University of Padova, Padua, Italy; ^4^Padova Neuroscience Center (PNC), University of Padova, Padua, Italy

**Keywords:** COVID-19 pandemic, lockdown, mental health – state of emotional and social well-being, resilience (psychological), personality, alexithymia (TAS-20)

## Abstract

Following the COVID-19 pandemic, many countries worldwide have put lockdowns in place to prevent the virus from spreading. Evidence shows that lockdown measures can affect mental health; it is, therefore, important to identify the psychological characteristics making individuals more vulnerable. The present study aimed, first, to identify, through a cluster analysis, the psychological attributes that characterize individuals with similar psychological responses to the COVID-19 home confinement; second, to investigate whether different psychological characteristics, such as personality traits, alexithymia, and resilience, specifically influence anxiety, stress, and depression, depending on the scope of the confinement. We analyzed data from 393 participants who completed an online survey on their experiences during two different phases of the Italian lockdown, characterized by more or less strict measures of confinement. Two clusters were identified which included participants reporting a better (+ER) and worse (−ER) emotional response to the lockdown, respectively. Individuals in the −ER group showed lower emotional stability, resilience, and higher alexithymia. Moreover, even if lifting part of the restrictions decreased psychological distress among all participants, a reduction in perceived stress was observed only among individuals with high resilience. Finally, personality traits, alexithymia, and resilience differently affected depression, anxiety, and stress. Our results suggest that different psychological interventions should be planned depending on the context: mental health professionals should focus on enhancing the individuals’ coping strategies to alleviate stress in emergencies, while long-term intervention aiming at alleviating anxiety and depressive symptoms should focus on alexithymic tendencies and personality constructs.

## Introduction

In early 2020 COVID-related restrictions forced people to stay home, in social isolation, blocking daily activities for months. This dramatic context triggered critical psychological consequences, such as anxiety, stress, depression, frustration, irritability, insomnia, post-traumatic stress symptoms, and anger (Brooks et al., 2020; Di Giuseppe et al., 2020; Franceschini et al., 2020; Salari et al., 2020). Studies from different countries showed that the incidence of these psychological problems was high everywhere: for example, surveys on Chinese respondents showed that almost 35% of the participants experienced psychological distress (Qiu et al., 2020) and that 2.9% scored above the cut-off for post-traumatic stress disorder (PTSD) and 9% scored at or above the clinical cut-off for depression (Tang et al., 2020). In another survey, including mostly participants from the United States and Israel, 22.2% of the population met the threshold for generalized anxiety, and 16.1% for generalized depression (Barzilay et al., 2020). In the Italian population, it was reported that around 20% of participants of a survey distributed during the first weeks of the lockdown experienced depression, anxiety, or high perceived stress, and 37% experienced post-traumatic stress symptoms (Rossi et al., 2020) while another study reported that 20% of the general population reported post-traumatic stress symptoms (Castelli et al., 2020). Similar percentages were reported in the general Italian population also by Mazza et al. (2020a) and Landi et al. (2020). A meta-analysis on the effects of the pandemic worldwide published in July 2020 indicated that the prevalence of stress was 29.6% (five studies, 9,074 participants), the prevalence of anxiety was 31.9% (17 studies, 63,439 participants), and that of depression was 33.7% (14 studies, 44,531 participants; Salari et al., 2020). Another systematic review confirmed high rates of symptoms of anxiety, depression, post-traumatic stress disorder, and stress in the general population during the COVID-19 pandemic in China, Spain, Italy, Iran, the United States, Turkey, Nepal, and Denmark (Xiong et al., 2020).

Among the psychological dimensions that mediate the relationship between stressors and mental health outcomes during lockdowns, an important role is played by individual differences in personality traits (Segerstrom and Smith, 2019), resilience and coping strategies (Serafini et al., 2020), and alexithymia (Hua et al., 2014). With regard to personality, one of the most accepted and used models is the five-factor model, or the big five (Goldberg, 1990), which includes emotional stability (also referred to as neuroticism), extraversion, openness, agreeableness, and conscientiousness. Openness, neuroticism, and extraversion, in particular, have been shown to influence the response to stress: for example, Oswald et al. (2006) found that self-reported Openness (the disposition to be inventive/curious, as opposed to being consistent/cautious) directly correlated with cortisol responses to stress. Schneider et al. (2012) found that during a stressful task, participants’ neuroticism (the disposition to be sensitive/nervous, as opposed to being resilient/confident) predicts higher negative affect, while openness and extraversion (being more outgoing/energetic, as opposed to being solitary/reserved) predict higher positive affect. It is, therefore, not surprising that individual differences in personality traits appear to play a role in the emotional response to the lockdown measures: higher neuroticism and lower extraversion have been associated with worse adaptation to lockdown (Carvalho et al., 2020; Morales-Vives et al., 2020) while Neuroticism has been found as a risk factor for psychological distress among Italian parents living with children (Mazza et al., 2020b).

As far as resilience and related coping strategies are concerned, studies show that they can be protective against the negative effects of stressors (Serafini et al., 2020). According to Fletcher and Sarkar (2013), resilience modulates how an event is evaluated, and based on this evaluation different coping strategies are engaged to manage the stress. With regard specifically to the role of resilience in the mental health outcome during the COVID-19 pandemic, Barzilay et al. (2020) provided evidence that a higher level of resilience was associated with lower COVID-19 related worries and a reduced level of anxiety and depression in both healthcare and non-healthcare professionals. Morales-Vives et al. (2020) supported this evidence, showing that people that best adapted to lockdown presented higher levels of resilience and successful coping.

Finally, evidence suggests that alexithymia, which describes a difficulty in identifying and describing subjective feelings and an externally oriented thinking (EOT) style (Sifneos, 1973), modulates the cortisol level in response to stress events (de Timary et al., 2008; Hua et al., 2014) and is a predictor for a high level of anxiety and depression (e.g., Honkalampi et al., 2000; Berardis et al., 2008; Fietz et al., 2018). Interestingly, it has been found that alexithymia has a mediator role in the association between COVID-19 pandemic exposure and PTSD and depressive symptoms (Tang et al., 2020).

Given the reviewed evidence, here we analyzed the data of an online survey evaluating the rate of stress, anxiety, and depression during the COVID-19 among the Italian population with two goals. *First*, the present study sought to investigate how personality traits, resilience, and alexithymia affected the level of anxiety, stress, and depression during COVID-19 home confinement. Using a bottom-up approach, we ran a cluster analysis on the individuals’ self-reported level of stress, anxiety, and depression to divide participants into groups with a similar emotional response to the lockdown. We then compared these groups to identify psychological attributes more common among individuals with similar responses to the lockdown. *Second*, we investigated whether different psychological characteristics influence mental health differently depending on the scope of the confinement: while it has been shown that alexithymia, resilience, and personality traits have a role on the effects of lockdown on mental health (Barzilay et al., 2020; Carvalho et al., 2020; Mazza et al., 2020b; Morales-Vives et al., 2020; Tang et al., 2020), no study has so far considered all these factors together.

We, therefore, asked participants to rate their experiences during two different phases of the Italian lockdown. The first phase (Phase 1) of the lockdown in Italy, from the 10th of March to the 3rd of May was characterized by the strict enforcement of tight rules, such as the absolute prohibition to leave one’s residence if not for health, work, or otherwise essential reasons. During the second phase of the lockdown (Phase 2), which started on the 5th of May, some of these restrictions were lifted allowing people to leave their houses again to visit families and to do physical activity, and some non-essential activities. The data analyzed here were collected through an online survey administered during the second week of Phase 2: participants rated their experiences during the two phases, i.e., recollecting their experiences during the last 2weeks of Phase 1, and reporting their evaluations of the first 2weeks of Phase 2.

Our first hypothesis was that individuals who showed a better adaptation during the lockdown would show lower levels of neuroticism and alexithymia, and a higher level of resilience compared to individuals who suffered a stronger impact of the lockdown on their mental health. In addition, we hypothesized that individuals with personal characteristics linked to higher adaptability, such as high resilience, openness, and low neuroticism, would particularly benefit from the partial lift of restrictions that marked the beginning of Phase 2 of the lockdown.

## Materials and Methods

The data used in this study was acquired as part of a bigger project investigating the effects of the COVID-19 lockdown (Cecchetto et al., 2021).

### Participants

The study protocol was approved by the Ethics Committee of the University of Padova and was conducted in accordance with the Declaration of Helsinki 2013. Data were collected anonymously through an online Survey on the Qualtrics XM Platform, shared *via* social media through a snowballing procedure in which participants were asked to invite friends to participate in the study. The required minimum sample size was set using Green’s rule of thumb (Green, 1991), which yielded to a minimum number of participants of 154; however, our main constrain was temporal as data was only collected from the 14th to the 19th of May 2020. The target of the survey were Italian residents 18 or more years old. All respondents read the consent form and explicitly agreed to participate before starting the survey. No compensation for participating in the study was given.

Six hundred thirty-five participants started the survey. One hundred ninety-four participants were excluded for not completing the survey, seven because of missing information (five because of missing information on their gender), five because of pregnancy, two because they reported having contracted COVID-19, and 23 because they spent part or all of the lockdown outside the Italian territory. Moreover, 11 participants were excluded because they reported currently having a diagnosed psychiatric disorder. The final sample comprised 393 participants.

### Measures

As described in Cecchetto et al. (2021), the online survey was composed of three parts. First, participants answered questions regarding socio-demographic information (age, gender, education, pregnancy, presence of pathologies, COVID-19 infection, occupational status before the lockdown), and filled in the Toronto Alexithymia Scale (TAS-20; Bressi et al., 1996), the Brief Resilient Coping Scale (BRCS, Kocalevent et al., 2017) and the 10-item personality inventory (TIPI, Gosling et al., 2003; Chiorri et al., 2015). The TAS-20 measures the general level of alexithymia. Each item is scored from 1 (strongly disagree) to 5 (strongly agree), for a maximum total of 100 and it includes three subscales: Difficulty in identifying feelings (DIF; *difficulty identifying feelings and distinguishing between emotional feelings and the bodily sensations of emotional arousal*; Parker et al., 2003), difficulty in describing feelings (DCF; *difficulty finding words to describe feelings to other*; Parker et al., 2003) and EOT (*externally-oriented style of thinking*; Parker et al., 2003). The international cut-off values are the following: 20–50 = non-alexithymic subjects; 51–60 = borderline alexithymic subjects; 61–100 = alexithymic subjects (Bressi et al., 1996). The BRCS is a four-item scale measuring adaptive coping strategies. Responses are collected on a 5-points Likert scale ranging from 1 = “does not describe me at all” to 5 = “describes me very well.” The sum score varies between 4 and 20 where higher scores indicate higher levels of resilience. The TIPI is a short self-report measure of the big five personality traits (openness, conscientiousness, extraversion, agreeableness, and emotional stability) in which each personality dimension is measured by two items. All items are rated on a 7-point Likert-type scale ranging from 1 (strongly disagree) to 7 (strongly agree).

In the second and third part of the survey, participants were asked to evaluate their level of well-being during the first and second phases of the lockdown, respectively. Participants filled in, for each phase, the Patient Health Questionnaire (PHQ-2; Kroenke et al., 2003), investigating depressive symptoms, the Generalized Anxiety Disorder scale (GAD-2; Kroenke et al., 2007), and the Perceived Stress Scale (PSS-10; Mondo et al., 2019). Participants were asked to fill the questionnaires referring to the last 2weeks of the lockdown Phase 1, and the first 2weeks of the lockdown Phase 2.

The PHQ-2 is a two-item screening tool that measures the frequency of depressed mood and anhedonia. Each item is scored from 0, “not at all,” to 3, “nearly every day.” A PHQ-2 ≥ 3 showed a sensitivity of 83% for major depression (Kroenke et al., 2003). The GAD-2 scale is composed of the first two items of the GAD-7 and it assesses core anxiety symptoms. Each item is scored from 0, “not at all,” to 3, “nearly every day.” Total scores range from 0 to 6 and 3 is considered the cut-off for clinically relevant anxiety symptoms (Kroenke et al., 2007). The PSS-10 is a 10-item scale measuring thoughts and feelings related to stressful events. It has six negatively- and four positively-stated items rated on a 5-point Likert scale ranging from 0, “never,” to 4, “very often.” Higher scores imply higher levels of perceived stress (Mondo et al., 2019) and the maximum possible score is 40.

### Statistical Analyses

Data were cleaned and analyzed using the software R (R Core Team, 2017). All continuous variables were centered and scaled. The dependent variables consisted of the GAD, PHQ, and PSS questionnaires scores.

First, cluster analysis was performed based on the similarities and differences in reported levels of anxiety, depression, and stress in the two phases with the “kmeans” function from the R default stats package. The best number of clusters was determined with “NbCluster” (Charrad et al., 2014), which tests 30 methods that vary the combinations of the number of clusters and distance measures for the *k*-means clustering. Cluster stability was estimated through a bootstrapping approach (100 iterations) with the “bootcluster” package (Yu, 2017). Descriptive analyses on the resulted clusters were run using *t*-tests (stats package; R Core Team, 2017), chi-square tests (chisq.test function of the stats package, the R Core Team, 2017), and *post hoc* of variables with more than two levels (chisq.multcomp function, RVAideMemoire package; Hervé and Hervé, 2020).

Second, for each dependent variable (PHQ, GAD, and PSS), linear mixed models (LMMs) were computed using the “lmer” function (lme4 package, Bates et al., 2015) and explored using the Anova function type three of the car package (Fox et al., 2019). The predictors consisted of the five personality traits (openness, conscientiousness, extraversion, agreeableness, and emotional stability), the three subscales of alexithymia (DIF, DDF, EOT), the level of resilience, age, gender, and occupational status before the lockdown. All of these predictors were analyzed in interaction with the lockdown phase (first or second). In addition, a random intercept for participant ID was added to account for within-subject measures. To ensure that each predictor improved the models’ fit, models were simplified using the “step” function (lmerTest package, Kuznetsova et al., 2017), which relies on the AIC criterion (Bolker et al., 2009). Factors that did not significantly improve the models’ fit were removed (Depression: the level of resilience, DDF, EOT, agreeableness, conscientiousness, emotional stability, gender, occupational status before the lockdown; Anxiety: the level of resilience, DDF, EOT, agreeableness, conscientiousness, age, gender, occupational status before the lockdown; Stress: DDF, openness, conscientiousness, extraversion, agreeableness, and emotional stability, gender, occupational status before the lockdown). AIC values of the initial and final models were calculated using the anova function (stats package, R Core Team, 2017). Collinearity between predictors was measured by calculating the Variance Inflation Factors (VIF) with the vif function of the car package (Fox et al., 2019). *Post hoc* tests of interactions including categorical factors were corrected using the Benjamini & Hochberg’s False Discovery Rate method (Benjamini and Hochberg, 1995), and interactions including continuous factors were analyzed according to Aiken & West’s method (Aiken et al., 1991).

## Results

### Sample Characteristics

The final sample is composed of 293 women and 100 males aged between 18 and 74 (mean = 35.00, SD = 13.50). They reported to be students (*n* = 115), full-time workers (*n* = 180), part-time workers (*n* = 57), and non-employed (retired or unemployed, *n* = 41) before the lockdown. Of these participants, 15.8% were alexithyimic and 84.2% not alexithyimic. The mean of the BRCS score was 13.1 (SD = 3.4). With regard to the PHQ and GAD questionnaire, during Phase 1, 31.6% were above the cut-off for depression and 28.0% were above the cut-off for anxiety, while during Phase 2, 21.1% were above the cut-off for depression and 20.6% were above the cut-off for anxiety. The mean score for PSS during Phase 1 was 18.7 (SD = 3.1) and during Phase 2 was 18.1 (SD = 3.0).

### Characterization of the Sample by Levels of Anxiety, Depression, and Stress

**Figure 1:**
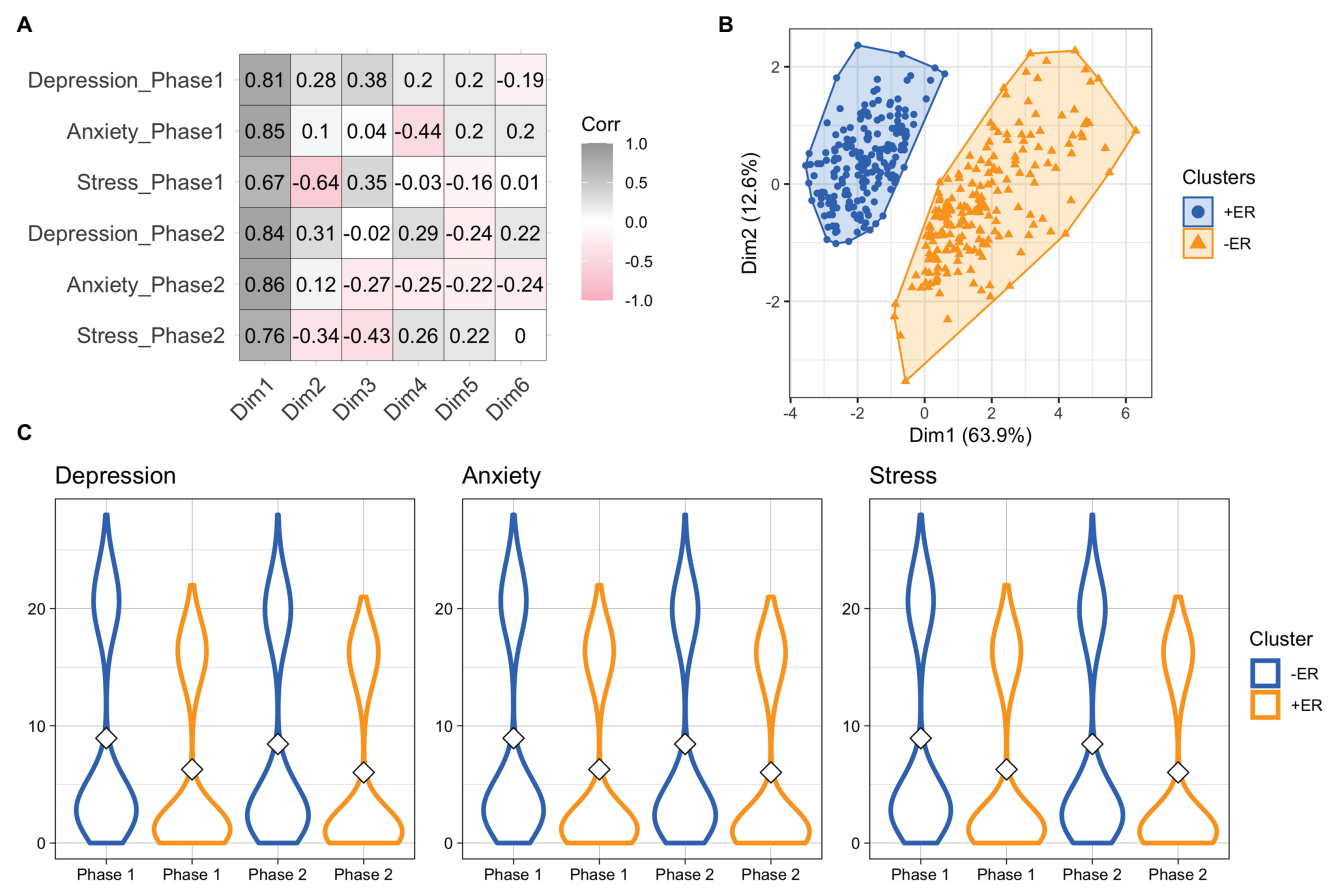
**(A)** Correlations between the six principal components with respect to changes in depression, anxiety, and stress in the two phases. Shades of gray indicate a positive correlation, whereas shades of red indicate negative correlations. White denotes no correlation. **(B)** Clusters of participants identified by *k*-means clustering. +ER = group of participants with positive emotional response; −ER = group of participants with negative emotional response. **(C)** Distribution of depression, anxiety, and stress by cluster and phase.

To better characterize our sample, a *k*-means algorithm was used to cluster participants based on the similarities and differences in the reported levels of anxiety, depression, and stress in the two phases (Figures 1A,B; see Supplementary Figure 1A in the Supplemental Material for a 3D representation of the clusters). The analysis showed that participants were clustered into two groups (bootstrapped stability = 0.93). Cluster 1 (*N* = 192) is composed by individuals with overall lower scores of depression, anxiety, and stress in both phases (cluster 1 means, Phase1: PHQ = 1.33, PSS = 16.41, GAD = 1.10; Phase 2, PHQ = 1.11, PSS = 16.04, GAD = 0.93). Cluster 2 (*N* = 201) is characterized by higher scores of depression, anxiety, and stress in both phases (cluster 2 means, Phase 1: PHQ = 2.99, PSS = 20.86, GAD = 2.97; Phase 2, PHQ = 2.57, PSS = 20.06, GAD = 2.72; Figure 1C). Since the cluster mean of cluster 1 is below cut-offs, we defined this group as reporting a better emotional response to the lockdown (positive emotional response, +ER) while cluster 2, which presents cluster means above cut-offs, includes participants with a negative emotional response to the lockdown (−ER).

Further investigations showed that the two groups significantly differ in age [*t*(381.75) = 3.06, *p* = 0.002], as group +ER includes older participants (mean = 37.12, SD = 14.14) than group −ER (mean = 32.97, SD = 12.66) and in distribution of women and men [*χ*^2^ (1) = 11.00, *p* < 0.001]: men were significantly more likely to be part of group +ER (67% of the total; *p* < 0.001) while the percentage of women did not significantly differ in the two groups (+ER, 42.7%, *p* = 0.14). The two groups also differ for the reported job before the lockdown [*χ*^2^ (3) = 90.90, *p* = 0.019]: as students were significantly more likely to be clustered in group −ER (61.74%, *p* = 0.019) while none of the other groups presents significant difference in the distribution of the two groups). Importantly, the two groups were also significantly different for the participants’ level of alexithymia [*t*(390.75) = −5.65, *p* < 0.001; group +ER, mean = 43.01, SD = 10.98; group −ER, mean = 50.00, SD = 11.78]. Considering the subscales of alexithymia, they were significantly different in the DIF [*t*(379.78) = −8.40, *p* < 0.001; group +ER, mean = 13.88, SD = 5.24; group −ER, mean = 18.88, SD = 6.53] and in the DDF [*t*(389.59) = −4.55, *p* < 0.001; group +ER, mean = 11.98, SD = 4.50; group −ER, mean = 14.04, SD = 4.44], but not in the EOT [*t*(390.22) = 1.35, *p* = 0.18; group +ER, mean = 17.15, SD = 4.20; group −ER, mean = 16.58, SD = 4.21]. Moreover, the two groups differ for resilience [*t*(389.54) = 2.40, *p* = 0.017; group +ER, mean = 13.47, SD = 3.36; group −ER, mean = 12.66, SD = 3.31]. There was no significant difference in any trait of personality except for emotional stability [*t*(390.68) = 7.16, *p* < 0.001; group +ER, mean = 4.59, SD = 1.28; group −ER, mean = 3.63, SD = 1.38].

### The Effects of Alexithymia, Resilience, and Personality on Individual Well-Being in the Two Phases of the Lockdown

To evaluate the specific effects of personality, resilience, and alexithymia dependent on the phase of the lockdown on stress, anxiety, and depression, we computed a LMM for each emotional measure. The final model investigating PHQ included phase, the DIF subscale of alexithymia, emotional stability, openness to experience, extraversion, and age, and ID as a random factor (initial AIC = 1,787.3, final AIC = 1,758.4, *p* = 0.86).

$$\begin{array}{l} \mathrm{PHQ}\sim \mathrm{Phase}+\mathrm{DIF}+\mathrm{Emotional Stability}+\mathrm{Extraversion}+\\ \mathrm{Openness To Experiences}+\mathrm{Age}+\mathrm{Phase}*\mathrm{Age}+\left(1\mathrm{|ID}\right). \end{array}$$

**Figure 2:**
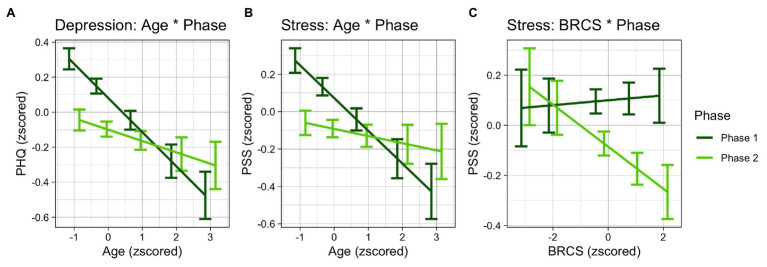
Fit lines of the interaction effects between **(A)** age and phase in depression; **(B)** age and phase in stress; **(C)** resilience and phase in stress.

Conditional *R*^2^ was equal to 0.74, and marginal *R*^2^ was equal to 0.31. Results showed a main effect for each predictor [Phase: *χ*^2^ (1) = 37.16, *p* < 0.001; TAS-DIF: *χ*^2^ (1) = 58.22, *p* < 0.001; Emotional stability: *χ*^2^ (1) = 18.73, *p* < 0.001; Extraversion: *χ*^2^ (1) = 9.43, *p* = 0.002; Openness to experiences: *χ*^2^ (1) = 6.71, *p* = 0.010; Age: *χ*^2^ (1) = 20.75, *p* < 0.001]. The main effect of phase showed an overall higher level of depression in Phase 1 than Phase 2. Results indicate that higher levels of depression were found among participants reporting higher scores in difficulties identifying feelings and who reported higher scores in openness to experience. On the other hand, lower levels of depression were found among participants who reported higher scores in emotional stability and extraversion. The interaction between age and phase [*χ*^2^ (1) = 13.02, *p* < 0.001; see Figure 2A] showed that during Phase 1, age indirectly correlated with depression [*t*(554.8) = −4.56, *p* < 0.001], while age did not affect depression scores in Phase 2 [*t*(554.78) = −1.52, *p* = 0.13], and that among younger participants, but not among older ones, depression was higher in Phase 1 than in Phase 2 [younger: *t*(393) = −6.86, *p* < 0.001; older: *t*(393) = −1.76, *p* = 0.080].

The final model investigating GAD included phase, DIF, and EOT of TAS, Emotional stability, Extraversion and openness to experience of TIPI, and ID as a random factor (initial AIC = 1,731.6, final AIC = 1,703.6, *p* = 0.76).

$$\begin{array}{l} \mathrm{GAD}\sim \mathrm{Phase}+\mathrm{DIF}+\mathrm{EOT}+\mathrm{Emotional Stability}+\\ \mathrm{Extraversion}+\mathrm{Openness To Experiences}+\left(1\mathrm{|ID}\right). \end{array}$$

Conditional *R*^2^ was equal to 0.75, and marginal *R*^2^ was equal to 0.37. Results showed a main effect for each predictor [Phase: *χ*^2^ (1) = 14.40, *p* < 0.001; DIF: *χ*^2^ (1) = 78.88, *p* < 0.001; EOT: *χ*^2^ (1) = 10.06, *p* = 0.002; Emotional stability: *χ*^2^ (1) = 43.92, *p* < 0.001; Extraversion: *χ*^2^ (1) = 4.91, *p* = 0.027; Openness to experiences: *χ*^2^ (1) = 7.60, *p* = 0.006] meaning that Phase 1 predicted a higher level of anxiety than Phase 2. Moreover, the two components of alexithymia showed an opposite effect: a higher level of difficulties to identify emotions predicted a higher level of anxiety but a higher level of external-oriented thinking predicted a lower level of anxiety. With respect to personality, higher levels of openness to experience predicted a higher level of anxiety while a higher level of emotional stability and extraversion predicted a lower level of anxiety.

The final model investigating PSS included phase, BRCS, DIF of TAS, EOS of TAS, age, and ID as random factors (initial AIC = 2,030.6, final AIC = 2,005.1, *p* = 0.68).

$$\begin{array}{l} \mathrm{PSS}\sim \mathrm{Phase}+\mathrm{BRCS}+\mathrm{DIF}+\mathrm{EOT}+\mathrm{Age}+\mathrm{Phase}:\mathrm{Age}+\\ \mathrm{Phase}:\mathrm{BRCS}+\left(1\;|\;\mathrm{ID}\right). \end{array}$$

Conditional *R*^2^ was equal to 0.55, and marginal *R*^2^ was equal to 0.55. Results revealed a main effect for phase [*χ*^2^ (1) = 17.03, *p* < 0.001], DIF [*χ*^2^ (1) = 67.09, *p* < 0.001], EOT [*χ*^2^ (1) = 10.59, *p* = 0.001], age [*χ*^2^ (1) = 13.99, *p* < 0.001] and a significant interaction between phase and BRCS [*χ*^2^ (1) = 3.87, *p* = 0.049], and between phase and age [*χ*^2^ (1) = 8.23, *p* = 0.004]. Results indicate that Phase 1 was a predictor of higher levels of stress as compared to Phase 2. As for anxiety, we found that a higher level of difficulties to identify emotions predicted a higher level of stress but a higher level of external-oriented thinking predicted a lower level of stress. As in depression, the interaction between age and phase specified that age predicted lower levels of stress in Phase 1 [*t*(638.43) = −3.74, *p* < 0.001; Figure 2B] but not in Phase 2 [*t*(638.43) = −0.82, *p* = 0.41], and that younger respondents had higher levels of stress in Phase 1 than in Phase 2 [*t*(393) = −4.95, *p* < 0.001], while this difference did not occur among older respondents [*t*(393) = −0.89, *p* = 0.38]. *Post hoc* on the interaction between phase and BRCS showed that resilience does not have effect in Phase 1 [*t*(616.89) = 0.20, *p* = 0.84; Figure 2C] while in Phase 2 it shows a trend of significance [*t*(616.89) = −1.72, *p* = 0.086], moreover it showed that in participants with higher levels of resilience [*t*(393) = −4.31, *p* < 0.001] the difference between phases is stronger than in participants with lower levels of resilience [*t*(393) = −1.52, *p* = 0.13].

## Discussion

The aim of the present study was twofold. First, through a cluster analysis, we characterized our sample of participants based on their level of anxiety, stress, and depression to unravel the psychological characteristics (personality traits, alexithymia, and resilience) of those who reported a stronger impact of the lockdown on mental health. Second, we explored more deeply the role of personality traits, alexithymia, and resilience on anxiety, stress, and depression in relation to the scope of the confinement.

### Characterization of the Sample Based on the Emotional Response to the Lockdown

Our results showed that individuals who had a better emotional response during the lockdown were characterized by high emotional stability, high resilience, and lower difficulties in identifying and describing feelings. In particular, the cluster analysis reported that our sample was best defined by two clusters, which included participants reporting a better emotional response to the lockdown (+ER, lower levels of depression, stress, and anxiety in both phases of the lockdown) and participants reporting negative effects on mental health (−ER, higher levels of depression, stress, and anxiety), respectively. Compared to the −ER group, +ER individuals showed higher scores of emotional stability, in line with previous results showing that individuals with higher emotional stability (lower neuroticism) reacted better to the lockdown (Mazza et al., 2020b; Morales-Vives et al., 2020). Moreover, they had a higher resilience score, which is in line with what has been already reported by Morales-Vives et al. (2020) and Barzilay et al. (2020), confirming that resilience can protect a person from negative emotional distress due to the pandemic. Finally, individuals of the −ER group, in contrast to +ER individuals, presented significantly higher levels of alexithymia, in particular in the difficulties in identifying and describing feelings subscales (DIF and DDF subscales of TAS questionnaire), a result in line with Tang et al. (2020) who reported a significant correlation between depression and PTSD symptoms and DIF and DDF subscales of alexithymia. In addition, the −ER cluster included younger individuals, a higher ratio of women, and students. This evidence confirms what was reported in the meta-analysis by Salari et al. (2020), i.e., that the prevalence of anxiety, depression, and stress during the COVID-19 pandemic is higher in women than in men and in individuals aged between 21 and 40. As suggested by Salari et al. (2020), women are in general more vulnerable to stress and post-traumatic stress disorder than men (Sareen et al., 2013; Lim et al., 2018). Moreover, younger people, even though they are less prone to undergo a severe illness caused by COVID-19, are probably more concerned over the future consequences of the pandemic than older people (Salari et al., 2020). This may contribute to the observed effect that students are more affected by the negative effects of lockdown than workers or unemployed individuals (the latter group included retired people, too); finally, students may have been more affected in terms of life changes, such as daily significant interactions, place of residence, and social life. Our results offer new evidence on the role of personality traits, resilience, and alexithymic tendencies in making individuals differently vulnerable to psychological distress during the lockdown.

### The Effects of the Severity of Imposed Restrictions on Mental Health

As already reported in Cecchetto et al. (2021), all the measures of psychological distress decreased significantly in Phase 2 as compared to Phase 1, showing that the loosening of the restrictions during Phase 2 helped people to better deal with home confinement. These results extended the findings of Morales-Vives et al. (2020) according to which the level of stress increased between the first and the third week of lockdown in Spain when restrictions became more severe. It is important to notice that, differently from Morales-Vives et al. (2020) who collected data from separated samples of participants across weeks, we reported measures from the same group of participants in the two phases. Avoiding most of the risks associated with interindividual differences, our within-subject comparison allows establishing a solid methodological background for a better understanding of the effects of personal features on psychological distress during the lockdown. Importantly, we observed that the severity of imposed restrictions had a different effect on mental health depending on the participants’ resilience and age. In particular, individuals with higher resilience, as compared to those with low resilience, exhibit a higher reduction in the stress level in Phase 2. This result may suggest that stronger resilience abilities helped individuals to recover in Phase 2 from the stress accumulated during Phase 1. Moreover, the contrast between strict and soft lockdown was particularly relevant for young people, since they reported a reduction of depression and stress in Phase 2 as compared to Phase 1.

### Effects of Psychological Traits on Depression, Anxiety, and Stress

The analysis of the effect of each psychological dimension separately on depression, anxiety, and stress levels, yielded interesting results. First, as to personality, we found that higher levels of emotional stability and extraversion, and lower levels of openness to experience predicted lower levels of depression and anxiety in both phases of the lockdown. At the same time, they did not affect stress levels. These results confirm previous studies showing that personality traits, and in particular extraversion and neuroticism, are strongly related to psychological well-being (see for example, Grant et al., 2009).

Second, resilience affected specifically the level of stress but not that of depression and anxiety. This suggests that coping strategies may be particularly useful in highly stressful situations and to cope with emergencies, as their effect on mental health consists of reducing the perceived stress, but not in protecting from long-term mood and anxiety disorders.

Third, regarding the role of alexithymia, we found that the DIF and EOT subscales impact emotional wellbeing. However, while higher levels of DIF predicts higher levels of depression, anxiety, and stress, higher levels of EOT seems to have a protective role for stress and anxiety. It has been reported that the sub-dimensions of alexithymia are probably related to different neural correlates, with the subscales measuring the difficulties identifying and describing feelings more related to each other and emotional distress (Parker et al., 2003; Eichmann et al., 2008; Pollatos et al., 2011). DIF has been frequently associated with increased negative affect and psychological distress (Liss et al., 2008; Li et al., 2015; Bagby et al., 2020). It is possible that the incapability of identifying feelings from bodily sensation may lead to difficulties in the evaluation and the regulation of emotions, making these individuals more vulnerable to chronic stress and mental illness (Preece et al., 2017; Fournier et al., 2020). On the other side, EOT has been associated with a reduced interest in viewing negative pictures (Wiebe et al., 2017) and with a utilitarian way of perception and avoid dealing with negative emotions (Taylor and Bagby, 2000), features that could have become useful in the current pandemic situation. In this framework, our findings suggest that while resilience can help cope with stress, alexithymia, and personality play a major role in influencing anxiety and depression.

### General Considerations

Two general considerations emerge from the present study. First, anxiety, depression, and stress were predicted by similar, though different, patterns of psychological dimensions. While personality traits influenced individual anxiety and depression, they did not affect the level of stress. On the other hand, resilience only affected stress but did not influence anxiety and depression. These results suggest that while personality impacts longer-term measures of emotional reaction, resilience may help only in modulating the perceived level of stress during such an exceptionally arousing event. This may offer a useful indication to mental health professionals as to the importance of different treatment goals depending on the context: during an emergency, it may be particularly useful in alleviating stress to enhance individuals’ coping strategies, while during long-term interventions focusing on anxiety and depressive symptoms a deeper work on personality constructs may be better indicated.

Second, unintuitively, alexithymic traits, and not the lack of resilience, may make individuals more vulnerable in extremely stressful circumstances. Indeed, resilience has only a marginal role in protecting individuals from the negative effects of lockdown and only in interaction with the loosening of restrictions. Resilience is considered a skill that is used to deal with and overcome stressful events and that helps not to develop maladaptive behaviors (Craparo et al., 2018) while, on the other hand, alexithymia is considered a stable personality trait (Luminet et al., 2001; Tolmunen et al., 2011). Probably for this reason, previous studies investigating the effects of the lockdown on psychological well-being seem to have focused more on resilience than alexithymia (Barzilay et al., 2020; Fullana et al., 2020; Landi et al., 2020, but see Tang et al., 2020). However, alexithymia can constitute a negative predictor for psychological treatment outcomes (Pinna et al., 2020) and recent treatment focus has been shifting on possible interventions on individuals with alexithymia, improving patients’ attentional control over interoceptive signals (Duquette, 2020). Our results suggest that interventions aiming at supporting the population during future lockdowns should therefore pay particular attention to individuals with alexithymic traits, as they may be less likely to seek support while being in need of it.

### Limitations

The present study presents some limitations. First, the study is not a longitudinal one and, although the same respondents provided the data regarding Phase 1 and Phase 2, the data has been collected relying on the abilities of the participants to remember how they had felt a week earlier. Future studies should implement a longitudinal design to confirm the effect of the scope of confinement on the variation of psychological well-being. Second, to comply with the exceptionality of the pandemic and the lockdown restrictions, the data were collected through self-report questionnaires presented online. This could have had an impact on the reported levels of anxiety, depression, and stress, which may not always converge with what would have resulted from an in-person assessment. In addition, we chose to administer brief versions of the questionnaires measuring depression and anxiety, to diminish the total number of survey questions that would have discourage individuals from participating. However, the selected tools were validated and have been commonly used. Third, the sample size is composed of an unequal number of women and men, as it has been recruited using non-probability sampling, which limits the generalizability of the findings. This is an issue familiar to many studies based on an online survey (Salari et al., 2020) that has been previously reported (Smith, 2008; Saleh and Bista, 2017). In the present study, we have tried to account for this issue by including gender as a fixed factor in the initial models. Fourth, our sample is rather small for an online survey; however, for the sake of the particular experimental design and the extraordinary historical moment, our survey was kept available only for 6days to be still able to collect reliable answers related to Phase 1 but at the same time to have people already felt the effects of Phase 2.

Despite these limitations, the current study provides valuable information on the factors influencing mental health during the COVID-19 lockdown, specifically in association with the influence of the scope of the confinement. We find that alexithymia and personality traits, together with age and gender, significantly impact the individuals’ levels of stress, anxiety, and depression, and that resilience is a protective factor specifically against stress, especially when lockdown restrictions are less strict. These indications expand the current knowledge of the influence of individual differences on emotional well-being during such an exceptionally stressful situation and can offer an indication of the kind of interventions that governments could put in place to limit the negative effects of confinement during future lockdowns.

## Data Availability Statement

The datasets presented in this study can be found in online repositories. The names of the repository/repositories and accession number(s) can be found below: the preprocessed datasets and the R code for the reported analyses can be found on the Open Science Framework database (https://osf.io/ukx5e/?view_only=746d4bfba444465e8d341bb63cf2eda9).

## Ethics Statement

The studies involving human participants were reviewed and approved by Ethics Committee of the University of Padova. The patients/participants provided their written informed consent to participate in this study.

## Author Contributions

MA, CC, and SO: development of the study concept and the study design, and data collection. SO and CC: data analysis under the supervision of MA, data interpretation, and manuscript writing. MA, CG, and SI: review and editing. MA and CG: supervision and project administration. All authors contributed to the article and approved the submitted version.

### Conflict of Interest

The authors declare that the research was conducted in the absence of any commercial or financial relationships that could be construed as a potential conflict of interest.
