# Supplementary material for: The Influence of Personality, Resilience, and Alexithymia on Mental Health During COVID-19 Pandemic
Source: Front Psychol. 2021 Feb 24;12:630751. doi: 10.3389/fpsyg.2021.630751 (PMC7943855; doi:10.3389/fpsyg.2021.630751)
Supplement: Supplementary file 1 [file Data_Sheet_1.zip › Figure 1A.html]

Figure 1A


# Figure 1A

Figure 1A: 3D representation of the two resulted clusters. PC1, PC2 and PC3 represents the first three components of the PCA analysis obtain from the k-means algorithm.

You must enable Javascript to view this page properly.
